# Case study: A patient with severe delusions who self-mutilates

**DOI:** 10.4102/sajpsychiatry.v26i0.1403

**Published:** 2020-06-25

**Authors:** Lesiba T. Lebelo, Gerhard P. Grobler

**Affiliations:** 1 Department of Psychiatry, Mamelodi Hospital, Pretoria, South Africa; 2 Department of Psychiatry, School of Medicine, University of Pretoria, Pretoria, South Africa

## Background and introduction

Although some overlapping features exist between self-injury and intention to die, there is growing recognition that non-suicidal self-injury (NSSI), including major self-mutilation (MSM), and suicidal behaviour are distinct entities as evidenced by their significance in terms of aetiology, psychiatric impairment, psychological function, method of self-harm and course or outcome between the two phenomena.^1^

We present a case of self-harm in a mental healthcare user diagnosed with schizophrenia to highlight the distinction made above.

## Case presentation

Mr X is a 38-year-old, unemployed, single male with no children and with an elementary level of education. This was his index presentation with a 4-year history characterised by ongoing persecutory delusions, as well as auditory hallucinations. He was brought to the Emergency Department by ambulance because he was found to be bleeding profusely from his scrotum in the toilet of a petrol filling station. He alleged that he had cut open his scrotum to remove his testicles before his ‘tormentors’ could do so. He stated clearly that he did not want to die because he valued his life. This was therefore not an attempt at suicide.

He was initially admitted to the urology ward and then referred to psychiatry. The multi-disciplinary team diagnosed him with and treated him for schizophrenia. He responded well to haloperidol 2.5 mg orally in the morning and 5 mg orally at night. A long-acting injectable antipsychotic, flupenthixol decanoate 20 mg intramuscular was also prescribed. No adverse effects were reported. Lorazepam was titrated downwards from 1 mg orally twice daily to 1 mg orally at night, and then stopped before he was discharged. Lansoprazole 30 mg daily orally, tramadol 50 mg three times daily orally and paracetamol 1 g orally were also prescribed as needed.

Upon discharge, on day 44 of the admission, the patient was symptom free with no psychotic or anxiety features.

The patient did not manifest any depressive symptoms throughout his hospitalisation, nor on his 4-week follow-up visit subsequent to discharge. He also demonstrated full and complete understanding that the voices, the self-conviction and his belief that people were coming to harm him were all part of his illness called schizophrenia. He also demonstrated full understanding that the belief of being harmed and people conspiring against him were also part of his schizophrenic illness that had been untreated for at least the past 4 years. With no negative emotion, he demonstrated intellectual understanding with unconditional acceptance of his illness. We emphasised to him that he must be consistent with medical check-ups at his local clinic as some other medical conditions can cause his illness to resurface. It was further emphasised to him that for as long as he took his treatment regularly and as prescribed the schizophrenia would be managed and controlled well. He agreed to stay away from all psychoactive substances. This user was amenable to following up with a clinical psychologist, an occupational therapist and a social worker.

He was followed up 1 month later and then referred to his local clinic for continuation of the prescribed treatment, appointments for continuation of psycho-education, counselling and relevant psycho-therapies. This patient responded well and remitted only on antipsychotic agents.

## Literature review and discussion

In a study of measurable variables, paranoia and auditory hallucinations, psychotic-like experience (PLE) and stressful life events all contributed to the patient causing self-harm. Compared to those without PLEs, the prevalence of NSSI was higher than those with PLEs.^1^

Psychotic-like experiences are highly prevalent in the general population, with figures of 20% or above being reported in some studies.^1^ Major self-mutilation (or NSSI) is a rare but potentially catastrophic complication of severe mental illness. Most people who inflict NSSI have a psychotic disorder, usually a schizophrenia spectrum psychosis. It is not known when in the course of psychotic illness, NSSI is most likely to occur.^2^ In general, schizophrenia is associated with worse social functional outcomes compared with other psychotic disorders, but the few studies that directly tested this assumption by comparing the longitudinal courses of social functioning in affective and non-affective psychoses have yielded conflicting findings.^3^

Cases of genital self-mutilation reported in the literature have been in patients with psychosis, including schizophrenia.^4^ Our own literature review found only a few case reports, published in 1974 (a female patient with schizophrenia and erotomania), 1986 (autocastration with biblical delusions) and in 1995. Greilsheimer writes that: ‘Men who intentionally mutilate or remove their own genitals are likely to be psychotic…’.^5^

The reason for presenting the case is that there was no similar case recorded in our country, using Google Scholar search engine database of at least the past 5 years, nor elsewhere when we searched using the following keywords: ‘Self-castration, non-suicidal self-injury and psychosis, self-castration due to psychosis’.

In the South African context, the promulgation of the *Traditional Health Practitioners Act* no. 35 of 2004 has become an important precipitant for the local review of the place of culture and religion/spirituality in secular areas such as health, mental health and spirituality.^6^ Our patient did not display delusions with religious or spiritual content. This particular patient was not practising any religion although he claims to believe in God. He emphasised that he was convinced by his delusions and hallucinations that some people known to him were conspiring to cut his scrotum and extract his testicles for some ritualistic practices. Their psychosis can eventually weaken their faith as they may think that they have been successfully bewitched and cursed even if they have been mentally stabilised.

Patients living with schizophrenia and who suffer persistently high levels of psychotic symptoms as well as poorer (psychosocial) functioning and lower self-esteem have higher severity of suicide behaviour.^7^ Even in first episode psychosis, one in 10 people engages in self-harm.^8^

It is important to take note of this case as it is the first of its kind and adds to existing knowledge in mental health that untreated and long-standing psychosis can result in the patient harming himself irreversibly such that they lose the capacity to reproduce.

Despite the vulnerable position of the testicles, testicular trauma is relatively uncommon. The mobility of the scrotum may be one reason, severe injury is rare. Given the importance of preserving fertility, traumatic injuries of the testicles deserve careful attention. Testicular injuries can be divided into three broad categories based on the mechanism of injury: (1) blunt trauma, (2) penetrating trauma and (3) degloving trauma. Such injuries are typically seen in males aged 15–40 years.

## Conclusion

Our patient was psychotic with auditory hallucinations, persecutory delusions and bizarre delusions which did not include religious delusions when he harmed himself. He was convinced that his ‘tormentors’ were listening to his thoughts and he consequently planned to cut open his scrotum to remove his testicles before they could do that to him. The main reason our patient injured himself was not to die but to relieve himself of the constant and increasing threats of being robbed of his testicles. It is important in the South African context to treat a psychiatric patient by using the multi-disciplinary team approach which is also holistic in nature and covers all aspects of mental healthcare service provision, including spirituality, as most citizens (92%) of South Africa expressed religious affiliation.^9^

Not all patients who harm themselves, even severely, are suicidal. Some just want to rid themselves of tormenting psychosis as in this case.
